# Analysis of the clinical characteristics of osteomyelitis in infants aged under 6 months

**DOI:** 10.1097/MD.0000000000049529

**Published:** 2026-06-26

**Authors:** Yu Ting Wang, Hai Ting Jia, Ming Zhu Lu, Jian Chen

**Affiliations:** a Department of Orthopedic Trauma Surgery, Children’s Hospital Affiliated to Shandong University (Jinan Children’s Hospital), Jinan, Shandong, China; b Department of Neonatal Intensive Care Unit, Children’s Hospital Affiliated to Shandong University (Jinan Children’s Hospital), Jinan, Shandong, China.

**Keywords:** clinical characteristics, infant, osteomyelitis, prognosis

## Abstract

This study aimed to summarize the clinical characteristics and prognosis of osteomyelitis in infants under 6 months of age. A retrospective analysis was conducted on the clinical data of 24 infants under 6 months of age diagnosed and treated for osteomyelitis between November 2017 and October 2024. This was a single-center study. General information, clinical manifestations, laboratory tests, treatments, and prognosis were summarized. Twenty-four infants (13 boys, 11 girls; age 0.5–5.6 months) were included. All presented with limb swelling, reduced movement, or pain on passive movement. Fever was initially present in 7 cases and developed early in 21 (87.5%). The femur (11/29, 37.9%) and tibia (7/29, 24.1%) were the most common infection sites. At admission, elevated WBC, C-reactive protein, and erythrocyte sedimentation rate were observed in 23 (95.8%), 22 (91.7%), and 19 (79.2%) infants, respectively. *Staphylococcus aureus* was the predominant pathogen (14/24, 58.3%). All received empirical antibiotics, adjusted based on culture results. Twenty underwent cortical window drainage. Follow-up of 21 infants (6 months to 8.5 years) revealed 1 case each of limb shortening, genu valgum, and restricted joint movement; the remaining 18 had normal limb function. Patients were stratified by prognosis for comparison. No significant differences were found in any variables between the 2 groups. Osteomyelitis in infants under 6 months of age lacks specific early clinical manifestations. Prompt laboratory and imaging evaluations are critical for early diagnosis and treatment. Overall, the prognosis is favorable.

## 1. Introduction

Osteomyelitis is a bacterial infection of the bone, typically resulting from hematogenous spread, with *Staphylococcus aureus* being the most common causative pathogen. It usually presents with pain, swelling, increased local skin temperature, and limited limb movement at the affected site.^[1–3]^ Based on the duration of symptoms, it is classified as acute (within 2 weeks), subacute (2 weeks to 3 months), or chronic (longer than 3 months).^[2]^ Osteomyelitis occurs more frequently in school-aged children, while it is relatively rare in infants. Infants aged under 6 months gain passive immunity through intrauterine exposure and breastfeeding, with a low incidence of osteomyelitis. Atypical clinical signs in these young infants often cause diagnostic delay and increase the risks of complications and long-term sequelae relative to older children. Currently, there are limited reports in the literature on osteomyelitis in infants, particularly those under 6 months of age. This study retrospectively analyzed the clinical data of 24 infants under 6 months of age diagnosed and treated for osteomyelitis between November 2017 and October 2024 to summarize their clinical characteristics and outcomes.

## 2. Materials and methods

### 2.1. Study subjects

This study selected 24 infants under 6 months of age who were diagnosed and treated for osteomyelitis between November 2017 and October 2024 as the study subjects. Inclusion criteria were as follows: diagnosis of osteomyelitis based on imaging studies, bacterial culture, and/or surgical and pathological findings; age < 6 months; and complete clinical data. Exclusion criteria included immune system diseases, hematological system diseases, bone tumors, open fractures, and osteomyelitis caused by mycobacteria or fungi. After admission, they underwent laboratory tests including complete blood count, C-reactive protein (CRP), erythrocyte sedimentation rate (ESR), and imaging examinations such as MRI.

This retrospective study was approved by the Ethics Committee of the authors’ affiliated institution, and the requirement for informed consent was waived due to the study’s retrospective nature and the use of anonymized data.

### 2.2. Research methods

The clinical data of infants under 6 months of age with osteomyelitis were collected and analyzed. The collected data included the following: general information (gender, age, course of disease, and length of hospital stay); clinical manifestations (initial symptoms, highest body temperature at onset, site of involvement, presence of concomitant arthritis, and whether adjacent epiphyses were affected); auxiliary examination results (white blood cell count [WBC; reference value 4–10 × 10^9^/L], CRP [reference value 0–10 mg/L], ESR [reference value 1–15 mm/h], identified pathogens, and imaging findings); and treatment details and prognosis.

Statistical analyses were performed using SPSS version 22.0 (International Business Machines Corporation, Armonk) on cases that completed follow-up. Patients were stratified by complication status. Because the small sample size precluded robust normality testing, a normal distribution was not assumed. Continuous variables are presented as median (IQR) and compared using the Mann–Whitney *U* test. Categorical variables are presented as frequency (%) and compared using the Fisher exact test. Statistical significance was set at *P* < .05 (two-tailed).

## 3. Results

### 3.1. General results

A total of 24 infants under 6 months of age with osteomyelitis were included in this study. Among them, there were 13 boys (54.2%) and 11 girls (45.8%). The median age at onset was 1.30 (0.80, 3.00) months, including 5 neonates (20.8%) and 19 non-neonates (79.2%). The median duration of illness before presentation was 4.00 (2.00, 10.00) days. Twenty-one cases (87.5%) were classified as acute infections, and 3 cases (12.5%) as subacute infections. The median hospital stay duration was 30.00 (25.00, 32.00) days.

### 3.2. Clinical characteristics

All patients presented with local swelling, reduced limb movement, or crying upon passive movement. Among them, 7 cases presented with fever as the initial symptom. A total of 21 cases (87.5%) developed fever during the early stage of the disease, with peak body temperatures ranging from 37.5°C to 40.3°C. Antibiotic therapy prior to admission was administered in 9 patients. Sites of involvement included proximal femur (5 cases), proximal tibia (4 cases), distal femur (3 cases), proximal humerus (1 case), distal humerus (1 case), proximal fibula (1 case), proximal radius (1 case), proximal ulna (1 case), metatarsal bone (1 case), and sternum (1 case). One case involved both the proximal tibia and distal femur simultaneously; 1 case involved both the distal and proximal humerus; 1 case involved both the proximal humerus and proximal tibia; 1 case involved both the distal and proximal femur; and 1 case involved both the distal tibia and fibula. The femur was the most commonly affected site (11/29, 37.9%), followed by the tibia (7/29, 24.1%; Table 1).

**Table 1:** General and clinical characteristics.

	Gender (F/M)	Age (mo)	Disease course (d)	Site	Complicated by arthritis	Involvement of adjacent epiphysis (Y/N)	Hospital stay (d)	Peak temperature at onset (°C)
1	F	0.8	4	Left proximal tibia, distal femur	Left knee arthritis	Y	91	38.5
2	M	0.5	8	Right proximal femur	Right knee arthritis	Y	32	38.2
3	F	0.5	2	Right proximal femur	Right knee arthritis	Y	29	37.9
4	F	1	6	Left proximal femur	Left knee arthritis	Y	48	39
5	M	1	3	Left proximal radius	Left elbow arthritis	N	29	39.2
6	F	1	3	Left distal femur	None	Y	25	No fever
7	M	1.3	2	Right proximal tibia	None	N	30	38.5
8	F	3	26	Right proximal humerus	Right shoulder arthritis	Y	32	No fever
9	F	1.6	1	Right proximal tibia	None	Y	25	38.7
10	F	0.8	1	Right proximal ulna	Right elbow arthritis	Y	18	38.8
11	F	3.3	6	Right distal tibia and fibula	None	N	22	37.5
12	F	4.7	4	Left distal humerus, proximal humerus	Left elbow arthritis	N	24	39
13	M	1.3	3	Right proximal femur	Right hip arthritis	N	26	38.5
14	F	5.6	1	Right proximal fibula	None	N	33	38.2
15	F	4.5	9	Left distal humerus	None	N	22	39
16	M	5	20	Right proximal femur	None	N	31	No fever
17	M	1.5	23	Right third metatarsal	None	Y	19	38.9
18	M	0.6	12	Left proximal tibia	Left knee arthritis	Y	31	38.6
19	M	1.3	12	Sternum	None	N	32	38.5
20	M	1.9	10	Right distal femur	Right knee arthritis	Y	25	38.6
21	M	1.3	10	Left proximal humerus, right proximal tibia	Left shoulder arthritis, right knee arthritis	Y	33	38.9
22	F	4.8	11	Right proximal tibia	None	N	32	40.3
23	M	1	7	Right distal femur	None	N	25	38.5
24	F	3	4	Right proximal femur, distal femur	None	N	33	39

### 3.3. Laboratory and imaging findings

Upon admission, elevated WBC counts were observed in 23 cases (95.8%), with a median initial WBC count of 14.44 (12.22, 18.40) × 10^9^/L. Elevated CRP levels were found in 22 cases (91.7%), with a median CRP level of 36.06 (22.87, 89.80) mg/L. Elevated ESR was noted in 19 cases (79.2%), with a median ESR level of 37.00 (20.00, 57.50) mm/h. Among the 24 patients, blood culture was performed in 19 patients, with 4 positive results (positive rate: 21.1%). Pus culture was performed in 21 patients, with 13 positive results (positive rate: 61.9%). Pathogens identified included methicillin-sensitive *S aureus* in 10 cases, methicillin-resistant *S aureus* in 4 cases, and *Salmonella enteritidis* in 1 case. Bacterial cultures were negative in 9 cases. All patients underwent magnetic resonance imaging (MRI) examination, which revealed abnormal signals in the medullary cavity of the affected site in all cases. Concomitant arthritis was present in 12 cases, including knee arthritis (7 cases), elbow arthritis (3 cases), shoulder arthritis (2 cases), and hip arthritis (1 case). Additionally, 12 cases showed abnormal signals in the adjacent epiphyses (Table 2).

**Table 2:** Laboratory and pathogen data.

	Pathogen	WBC (×10^9^/L)	CRP (mg/L)	ESR (mm/h)
1	MSSA	17.52	55.61	2
2	MRSA	18.58	13.95	47
3	None	13.53	109	42
4	None	11.13	23	4
5	MSSA	13.57	36.33	5
6	None	14.1	27.7	2
7	MSSA	14.45	50.65	31
8	None	10.5	11.8	25
9	None	18.27	108.1	75
10	MSSA	18.51	112	112
11	MSSA	16.09	12.06	18
12	MSSA	8.38	34.84	20
13	MSSA	28.94	102.32	50
14	None	14.42	58.3	37
15	None	12.3	2.07	65
16	None	14.33	23.49	22
17	MSSA	11.04	<0.499	6
18	MRSA	17.04	143.06	44
19	None	29.19	45.38	92
20	MSSA	12.85	48.44	69
21	*S enteritidis*	11.87	35.29	44
22	MSSA	21.84	89.6	42
23	MRSA	20.89	10.3	22
24	MRSA	24.97	98.49	111

CRP = C-reactive protein, ESR = erythrocyte sedimentation rate, MRSA = methicillin-resistant *Staphylococcus aureus*, MSSA = methicillin-sensitive *Staphylococcus aureus*, WBC = white blood cell count.

### 3.4. Treatment and prognosis

All patients received empirical antibiotic therapy upon admission, which was subsequently adjusted based on bacterial culture and drug sensitivity test results. The total duration of antibiotic therapy was generally 6 weeks (intravenous followed by oral). Surgical intervention was performed for patients who showed no significant improvement after antibiotic therapy, had further elevated inflammatory markers, or had formed large abscesses. In this study, 20 patients underwent surgery, all receiving cortical fenestration and drainage. Postoperative wound healing was good in all surgical cases.

As of the follow-up in May 2025, 3 cases were lost to follow-up. The remaining 21 cases were followed up for periods ranging from 6 months to 8.5 years. Among them, 1 case developed limb shortening, 1 case developed genu valgum, and 1 case exhibited limited joint mobility. The remaining 18 cases showed good limb function.

### 3.5. Impact on prognosis

Complete case analysis was adopted for handling lost data. Of the 24 initial cases, 3 (12.5%) were excluded due to loss to follow-up, and the final analysis included 21 cases with clear prognostic outcomes (18 favorable, 3 unfavorable).

There were no statistically significant differences between the 2 groups with respect to any of the continuous variables examined, including WBC, ESR, CRP, length of hospital stay, and duration of onset (all *P* > .05). Similarly, no statistically significant differences were found in the categorical variables: the proportion of patients with arthritis (50.0% vs 66.7%, *P* = 1.000), epiphyseal involvement (44.4% vs 66.7%, *P* = 1.000), and surgery (66.7% vs 100%, *P* = .538) did not differ significantly between the 2 groups (Table 3).

**Table 3:** Baseline characteristics and prognostic comparison.

Variable	Group without complications (n = 18)	Group with complications (n = 3)	*P* value
WBC (×10^9^/L)	14.42 (13.53, 18.27)	17.04 (11.87, 20.89)	.865
CRP (mg/L)	48.44 (23.49, 102.32)	35.29 (10.30, 143.06)	1.000
ESR (mm/h)	37 (20, 65)	44 (22, 44)	.744
Hospital stay (d)	29 (25, 32)	31 (25, 33)	.500
Onset duration (d)	4 (2, 8)	10 (7, 12)	.125
Arthritis (Y)	9 (50.0%)	2 (66.7%)	1.000
Epiphyseal involvement (Y)	8 (44.4%)	2 (66.7%)	1.000
Surgery (Y)	12 (66.7%)	3 (100%)	.538

CRP = C-reactive protein, ESR = erythrocyte sedimentation rate, WBC = white blood cell count.

## 4. Discussion

Osteomyelitis occurs more frequently in school-aged children, with the incidence rate in boys typically being about twice that in girls.^[2,4]^ In this study, the male-to-female ratio in infants under 6 months was close to 1.2:1. This discrepancy may stem from 2 reasons: on one hand, the sample size of this study is relatively small; on the other hand, some studies suggest that the higher prevalence in boys might be associated with a greater susceptibility to trauma.^[5]^ However, in this study, all infants were under 6 months of age, in the early infant stage, where the risk of trauma exposure is relatively low. Therefore, the influence of trauma on the gender difference in incidence might not be significant in this specific age group. According to the duration of symptoms at presentation, most cases in this study were classified as acute infections. The most common sites of osteomyelitis are the femur and tibia, and the most frequent causative pathogen is *S aureus*, accounting for approximately 22.0% to 77.6% of cases.^[1,2]^ This aligns with the findings of this study, where osteomyelitis in infants under 6 months most commonly affected the femur (11/29, 37.9%) and tibia (7/29, 24.1%).

The most common pathogen was *S aureus* (14/24, 58.3%), of which methicillin-resistant *S aureus* accounted for 28.6% (4/14) of the *S aureus* isolates. This is consistent with the study by Mohamad et al, which reported that the most common pathogen in infants younger than 6 months was methicillin-sensitive *S aureus*, whereas *Kingella kingae* was the most common pathogen in infants aged 6 to 12 months.^[6]^ Some literaturereports that Group B Streptococcus and gram-negative bacilli are common pathogens in neonates and young infants, particularly those under 3 months of age, accounting for about 60% of cases in such infants.^[4,7]^ Group B Streptococcus was not identified in this study, but 1 case was infected with *S enteritidis*. The 9 culture-negative cases may be related to antibiotic use before admission, low microbial load, or difficulties in culturing certain pathogens.^[8–10]^

The early clinical manifestations of osteomyelitis in infants are often atypical and may include only lethargy, irritability, or poor feeding, which can easily be overlooked, potentially leading to a delayed diagnosis.^[11]^ Literature reports indicate that common clinical manifestations in infants include local swelling, refusal to move the limb, increased local skin temperature, and fever.^[12]^ However, due to the relatively thick subcutaneous fat in infants, detecting swelling and increased skin temperature at the affected site can be challenging in clinical practice. In this study, patients presented with local limb swelling, reduced movement, or fever. Local swelling was the initial symptom in 66.7% (16/24) of cases, whereas fever was the initial symptom in 29.2% (7/24). Concomitant arthritis was observed in 54.2% of the infants, which might be related to the communication between metaphyseal and epiphyseal vessels, facilitating the spread of infection from the metaphysis to the epiphysis, potentially destroying the epiphysis and subsequently extending into the joint.^[13]^

Common laboratory tests for diagnosing osteomyelitis include complete blood count, CRP, and ESR. Among these, CRP and ESR have been proven to hold the greatest clinical value in assessing acute osteomyelitis.^[2]^ The WBC count is rarely elevated in neonates with osteomyelitis but is usually elevated in infants, young children, and older patients.^[14]^ CRP typically rises rapidly in the early stages of infection and generally normalizes within 7 days of effective treatment, thus playing an important role in monitoring the condition during the acute hospitalization period.^[15]^ The sensitivity of ESR alone for diagnosing osteomyelitis is approximately 79%, but when combined with WBC count, clinical symptoms such as high fever, and swelling/pain at the site of involvement, the sensitivity can reach 98%.^[2,16]^ ESR usually decreases slowly, requiring 2 to 3 weeks of treatment to normalize.^[2,16]^ In this study, WBC counts were elevated to varying degrees upon admission in 95.8% of patients, CRP levels were elevated in 91.7%, and ESR levels were elevated in 79.2%. Common imaging studies for diagnosing osteomyelitis include X-ray, ultrasound, and MRI. The primary role of X-ray in the initial phase is to exclude other conditions, as bony changes usually become apparent only after about 2 weeks of illness. Ultrasound is simple, safe, and can detect soft tissue abscesses and the presence of septic arthritis.^[17]^ MRI is the most sensitive imaging modality for diagnosing osteomyelitis, allowing visualization of abnormal signals in the medullary cavity or epiphysis.^[11]^

Treatment for osteomyelitis includes antibiotic therapy and surgical intervention. Antibiotic therapy is the cornerstone of osteomyelitis treatment. Empirical antibiotic therapy is initiated when the pathogen is unknown and is later adjusted based on antimicrobial susceptibility testing results. In recent years, early transition to oral antibiotic therapy for osteomyelitis has become increasingly common.^[4]^ A 2013 systematic review indicated that for uncomplicated acute hematogenous osteomyelitis, an early switch to oral antibiotics (after 3–4 days of intravenous therapy, total duration 3 weeks) had comparable efficacy to longer regimens, although this study was not applicable to neonates.^[18]^ Although the European Society for Paediatric Infectious Diseases suggests considering shorter courses for older children, it still recommends at least 2 to 3 weeks of intravenous therapy for infants, with a total antibiotic course of 4 to 6 weeks.^[19]^ In this study, the 24 infants received intravenous antibiotics for approximately 4 weeks on average, with a total course generally around 6 weeks. All patients eventually improved and were discharged. Surgical intervention is recommended when there is no significant clinical improvement after 2 to 4 days of antibiotic therapy or when imaging suggests abscess formation.^[20]^ Because of the fragile and soft cortical bone and the loose periosteum in young infants, abscesses can spread easily.^[1]^ Therefore, for osteomyelitis in young infants, the timing of surgery should be assessed promptly to avoid delays in treatment. In this study, 20 cases (83.3%) underwent cortical fenestration and drainage surgery. During follow-up, apart from 3 cases that developed limb shortening, genu valgum, and limited joint mobility, respectively, and 3 children lost to follow-up, the remaining surgical cases exhibited good limb function. The 4 cases treated solely with antibiotics also showed no adverse outcomes. Conservative cases had milder symptoms and no sequelae, whereas surgical cases had more severe involvement but similar favorable long-term outcomes.

This study has certain limitations. First, it is a retrospective single-center study with selection bias, which may affect the generalizability of the research results. Second, the sample size is small, and it is necessary to further collect cases to expand the sample size in the future. Subgroup analysis was not performed due to the small neonate sample and insufficient statistical power. We plan to conduct a multicenter collaboration to enlarge the sample size of neonates, which will provide sufficient statistical power for subgroup analyses in a follow-up study. Finally, the small sample size, particularly the low number of poor-prognosis events (n = 3), precluded multivariate analysis. Therefore, the independent predictors of prognosis need to be further validated in larger cohort studies.

In summary, osteomyelitis in infants under 6 months of age has the following clinical characteristics: comparable incidence between males and females; predominantly acute and subacute infections; common involvement of the femur and tibia; *S aureus* as the most frequent pathogen; early clinical manifestations are often nonspecific and primarily include local swelling at the affected site, reduced limb movement, or crying upon passive movement; WBC count, CRP, and ESR are usually elevated. The total recommended antibiotic course is 4 to 6 weeks, including at least 2 to 3 weeks of intravenous therapy. Because of the loose periosteum in young infants, abscesses spread easily, necessitating timely assessment for surgical intervention to avoid delayed treatment. With prompt and appropriate treatment, a majority of pediatric patients with juvenile osteomyelitis show a relatively positive prognosis.

## Author contributions

**Conceptualization:** Yu Ting Wang, Hai Ting Jia, Jian Chen.

**Data curation:** Yu Ting Wang, Jian Chen.

**Formal analysis:** Yu Ting Wang.

**Funding acquisition:** Jian Chen.

**Investigation:** Yu Ting Wang, Hai Ting Jia, Jian Chen.

**Methodology:** Hai Ting Jia, Jian Chen.

**Supervision:** Hai Ting Jia.

**Writing – original draft:** Yu Ting Wang, Hai Ting Jia.

**Writing – review & editing:** Hai Ting Jia, Ming Zhu Lu, Jian Chen.

## References

[1] Branco J Duarte M Norte S. Osteoarticular infections in infants under 3 months of age. Pediatr Int. 2022;64:e15212. 35938592 10.1111/ped.15212

[2] Funk SS Copley LA. Acute hematogenous osteomyelitis in children: pathogenesis, diagnosis, and treatment. Orthop Clin North Am. 2017;48:199–208. 28336042 10.1016/j.ocl.2016.12.007

[3] Peltola H Pääkkönen M. Acute osteomyelitis in children. N Engl J Med. 2014;370:352–60. 24450893 10.1056/NEJMra1213956

[4] Diamond S Vallejo JG McNeil JC. Microbiology and treatment outcomes of community-acquired hematogenous osteoarticular infections in infants ≤12 months of age. J Pediatr. 2022;241:242–6.e1. 34626668 10.1016/j.jpeds.2021.09.057

[5] Malcius D Trumpulyte G Barauskas V Kilda A. Two decades of acute hematogenous osteomyelitis in children: are there any changes? Pediatr Surg Int. 2005;21:356–9. 15834576 10.1007/s00383-005-1432-7

[6] Mohamad M Steiger C Spyropoulou V. Clinical, biological and bacteriological characteristics of osteoarticular infections in infants less than 12 months of age. Future Microbiol. 2021;16:389–97. 33847142 10.2217/fmb-2020-0070

[7] Offiah AC. Acute osteomyelitis, septic arthritis and discitis: differences between neonates and older children. Eur J Radiol. 2006;60:221–32. 16971078 10.1016/j.ejrad.2006.07.016

[8] Klosterman MM Villani MC Hamilton EC Jo C Copley LA. Primary septic arthritis in children demonstrates presumed and confirmed varieties which require age-specific evaluation and treatment strategies. J Pediatr Orthop. 2022;42:e27–33. 34560764 10.1097/BPO.0000000000001976

[9] Chen JA Lin HC Wei HM. Clinical characteristics and outcomes of culture-negative versus culture-positive osteomyelitis in children treated at a tertiary hospital in central Taiwan. J Microbiol Immunol Infect. 2021;54:1061–9. 32891539 10.1016/j.jmii.2020.08.005

[10] Özcan C Çamur S Shatat KMI Kemah B Söylemez MS Sağlam N. Comparison of clinical features and serum parameters of culture-positive children with culture-negative children in septic arthritis and acute osteomyelitis. Acta Chir Orthop Traumatol Cech. 2021;88:131–6. 33960926

[11] Dormans JP Drummond DS. Pediatric hematogenous osteomyelitis: new trends in presentation, diagnosis, and treatment. J Am Acad Orthop Surg. 1994;2:333–41. 10709026 10.5435/00124635-199411000-00005

[12] Sun K Zhang C Mao Z. Clinical characteristics of neonatal and infant osteomyelitis and septic arthritis: a multicenter retrospective study. J Pediatr (Rio J). 2024;100:430–7. 38642591 10.1016/j.jped.2024.03.003 PMC11331240

[13] Roversi M Chiappini E Toniolo RM. Neonatal osteomyelitis: an Italian multicentre report of 22 cases and comparison with the inherent literature. J Perinatol. 2021;41:1293–303. 33686117 10.1038/s41372-021-00956-4

[14] Kang SN Sanghera T Mangwani J Paterson JMH Ramachandran M. The management of septic arthritis in children: systematic review of the English language literature. J Bone Joint Surg Br. 2009;91:1127–33. 19721035 10.1302/0301-620X.91B9.22530

[15] Unkila-Kallio L Kallio MJ Peltola H. The usefulness of C-reactive protein levels in the identification of concurrent septic arthritis in children who have acute hematogenous osteomyelitis. A comparison with the usefulness of the erythrocyte sedimentation rate and the white blood-cell count. J Bone Joint Surg Am. 1994;76:848–53. 8200891 10.2106/00004623-199406000-00008

[16] Gigante A Coppa V Marinelli M Giampaolini N Falcioni D Specchia N. Acute osteomyelitis and septic arthritis in children: a systematic review of systematic reviews. Eur Rev Med Pharmacol Sci. 2019;23:145–58. 30977881 10.26355/eurrev_201904_17484

[17] Sendi P Kaempfen A Uçkay I Meier R. Bone and joint infections of the hand. Clin Microbiol Infect. 2020;26:848–56. 31917233 10.1016/j.cmi.2019.12.007

[18] Howard-Jones AR Isaacs D. Systematic review of duration and choice of systemic antibiotic therapy for acute haematogenous bacterial osteomyelitis in children. J Paediatr Child Health. 2013;49:760–8. 23745943 10.1111/jpc.12251

[19] Saavedra-Lozano J Falup-Pecurariu O Faust SN. Bone and joint infections. Pediatr Infect Dis J. 2017;36:788–99. 28708801 10.1097/INF.0000000000001635

[20] Woods CR Bradley JS Chatterjee A. Clinical practice guideline by the Pediatric Infectious Diseases Society and the Infectious Diseases Society of America: 2021 guideline on diagnosis and management of acute hematogenous osteomyelitis in pediatrics. J Pediatric Infect Dis Soc. 2021;10:801–44. 34350458 10.1093/jpids/piab027

